# Ever more parents in polyamorous families: A new materialist typology of parenting practices and division of work

**DOI:** 10.1177/13634607211037481

**Published:** 2021-09-16

**Authors:** Cornelia Schadler

**Affiliations:** 27258 University of Vienna, Wien, Austria

**Keywords:** Polyamory, parenthood, care work, division of work, new materialism

## Abstract

An analysis of parents that are a part of polyamorous networks—networks of three, four, or even more residential or highly available parents—shows three types of parenting practices: poly-nuclear, hierarchical, and egalitarian parenting. Especially, the hierarchical and egalitarian parenting practices show novel divisions of care work and a transgression of gender norms. However, in-depth new materialist analysis of qualitative interviews also shows how parents are, in specific situations, pushed toward standard family models and thus unintentionally maintain traditional family structures and gender roles.

## Introduction

Parenting research has, for decades, only included two parents, and for most parts focused on heterosexual, monogamous couples of a mother and a father (Guzzo and Hayford, 2020; Reczek, 2020). More than two parents were sometimes included in research of step-families, when separated parents repartner (Raley and Sweeney, 2020), or when LGBT couples deal with a third party to reach their status of parenthood (Reczek, 2020). In my research, I talked to parents who form groups of three, four, or more people who parent children together. They identified as members of consensual non-monogamous relationships, such as polyamorous, open, or relationship anarchist networks. Their practices and solutions did not fit to current definitions and typologies in parenting research. Resembling the experiences of many of these parents in their everyday reality, this group is overlooked in research on parenting and families (Reczek, 2020) and in research on non-monogamous relationships (Klesse, 2019). Only a few pioneering studies discussed the role of polyamorous parents (Pallotta-Chiarolli, 2010; Raab, 2018; Sheff, 2011).

In Germany and Austria, where this study was conducted, polyamorous families have no legal status. Custody laws in Germany and Austria are not designed to include more than two parents. Furthermore, polyamorous living arrangements are not represented in censuses and major surveys. However, polyamorous and polygamous living arrangements are considered to be the next important stage in the civil rights movement for legal recognition (Otter, 2016). These developments make it even more important to gather basic knowledge about this increasingly visible form of parenting. How do current non-monogamous networks parent their children? How do they divide work? What are their solutions for everyday processes?

This article provides two major findings: First, a typology of polyamorous parenting practices and second, an illustration of lived realities that, at times, do not fit this typology and the related self-identifications of the interviewed. A new materialist analysis helped to simultaneously identify the boundaries of parenting types and the dissolution of these boundaries in specific situations. My research found that parenting relationships are organized around three types of polyamorous networks: (a) poly-nuclear parenting (simulating two-parent families with biological and legal children), (b) hierarchical parenting (a network of at least three parents that differ on their degree of involvement), and (c) egalitarian parenting (where three or more members of a polyamorous network are perceived as equal parents). All three types of parenting in non-monogamous relationships are characterized by specific practices that are discussed in the findings. However, in the last section, I take a closer look at specific everyday practices, and it shows that parents do not always stick to one parenting type (even when they highly identify with a specific model of parenting), but are pushed and pulled through all of these parenting types over time, for example, when structural arrangements (work and school) change, or when a situation leaves no other choice.

### Research on polyamorous parents

To date, only a few projects have been concerned with the practices of parenting in polyamorous networks (Klesse, 2019). Sheff (2010, 2011) interviewed 71 people living in polyamorous families. Her research focused on the resources and challenges faced by polyamorous families such as non-visibility, a lack of legal status, the stigmatization of polyamorous practices, and the families’ relationship to the family of origin. Pallotta-Chiarolli (2010) has developed the notion of “border families” because polyamorous families exist within the mainstream norms of family while also deviating from them at the same time (see also the last section of this article). For her, this position within society allows her informants a specific view on norms because they are constantly navigating the borders of these norms. Her focus on the perception of polyamorous families in the Australian school system shows, how diverse family forms are silenced and made non-visible within the school system (Pallotta-Chiarolli, 2010). Raab (2018) investigated polyamorous families, some with children, and their division of (domestic) labor. Many polyamorous families claim to transgress traditional gender norms. However, in Raabs data only some truly transgress societal norms of gender roles and in many families (cis) women were responsible for domestic work and the care of children. Nevertheless, a few polyamorous networks managed to redistribute financial responsibilities and care work equally or against the traditional norm (Raab, 2018). The following paper will show that the processes discussed in this short review section may be related to specific family models.

## Method

The empirical data was gathered during the project “Family as a nexus of material-discursive practices” funded by an Erwin Schrödinger Fellowship of the Austrian Science Fund. The project’s main research question concerns the situative research practices that shape family boundaries and how differences between members and non-members of families emerge. One specific form of relationship I investigated in this project was polyamorous networks. I interviewed at least one member of 25 networks who identified as polyamorous. Informants were gathered through email lists of polyamorous communities and through word of mouth (informants I interviewed provided us with the contact details of other informants). Knowledge of this family form is too scarce to rely on a specific form of sampling beforehand; therefore, my aim was to interview as many informants I could get until a number of about 25 networks was reached. Thirteen of the interviewed networks have biological, legal, or social children. These interviews provide the basis for this article. The interviews with at least one person of these thirteen polyamorous networks with children are the basis for this article. In three occasions, I was able to interview more than one parent. In terms of socio-economic status, the sample is comprised mostly of people with higher education. The informants live all over Austria and Germany, in urban and rural areas. After a first reading of the interviews, if possible, I gathered additional data on issues or things mentioned in the interview. For example, when an informant mentions a specific book as helpful or important to her/him, I also included this book into my corpus of data. Subsequently, I included data from podcasts, newspaper pieces, websites, and books.

The study was conducted from the perspective of new materialism (Barad, 2007; Braidotti, 2002; Haraway, 2008; for an overview see Dolphijn and Tuin, 2012), which promises a complex analysis of qualitative data (Authors own; XXXX). New materialisms do not assume that any entity—such as values, structures, bodies, or meanings—precedes a specific process. Rather, they are theorized as being defined within a specific process. In the case of families, the consequence is that no specific structure or meaning can define the boundaries of family on its own, but the structure and meaning of family are produced within specific practices, along with many other entities that gain visibility from this perspective. For this reason, new materialist research aims to describe the practices, and hence the material and discursive processes, that form the boundaries of a specific relationship, in this case between polyamorous parents and their children. The ethnographic text of new materialist research therefore focuses on figurations, their boundaries, and entities configured within these boundaries (Haraway, 1992, 2008). Consequently, the complex interplay of participants, activities, identities, values, structures, and so on is explored, but no causal explanations can be drawn.

**Table:** 

Informant	Parenting practice	Parental status
Bernhard	Poly-nuclear parent	Biolegal and main parent
Clemens	Egalitarian parenting	Social and main parent
Daniela, Daniel	Hierarchical parenting	Co- and social parent, biolegal and main parent
Fiona, Flora, Fabian, Finn	Egalitarian parenting	Biolegal and main parent, social and main parent, social and main parent
Gerda	Egalitarian parenting	Social and main parent
Henry	Hierarchical parenting	Social and co-parent
Iwan	Egalitarian parenting	Social and main parent
Jana	Poly-nuclear parenting	Biolegal and main parent
Leo	Poly-nuclear parenting	Biolegal and main parent
Nena	Hierarchical parenting	Co- and social parent
Onna	Hierarchical parenting	Co- and social parent
Roger	Egalitarian parenting	Biolegal and main parent
Tori, Tina	Hierarchical parenting	Biolegal and main parent, legal and main parent

The method of analysis is based on a detailed analysis of a small number of cases (Schadler, 2019). It consists of a process of referencing activities and participants of practices in order to establish the practices the parents are a part of, as well as the practices that mark the boundaries between different forms of parenting. In this method of analysis a few sentences can produce numerous pages of references toward activities and human and non-human participants of activities. Recurring sets of references are grouped into practices and included into an ethnographic description of a set of practices.

It is important that, from a new materialist perspective, to become recognizable and visible, for example as a specific subform of family, boundaries must be drawn and reconfigured. Researcher can be a part of this process, in the case of this study, by co-creating a typology with the data. The empirical analysis of interviews is intended to rebuild the practices that the informants are a part of by making references to these material and discursive environments (Schadler, 2019). For this purpose, only a small amount of cases is necessary.

### The Central European context and the basic living conditions of multi-parent families

Parents in my sample live in Germany or Austria. Both countries are welfare states that provide public health care for their residents. Therefore, the costs of the child’s health care, such as the birth, visits to a pediatrician, or hospital bills are covered by these public insurances. Legal parents of children receive child benefits of €190 in Germany and €170 in Austria on a monthly, per-child basis. Parents on parental leave receive an income replacement of 65% of their income in Germany and up to €2000 a month in Austria, in both cases for a year after the birth of a child. Depending on regional policies, public daycare is free or reasonably priced in both countries. Also, both countries have excellent public schools. Therefore many of the costs involved in having and raising children, which parents in other countries have to pay for (Pallotta-Chiarolli, 2010), are covered for parents in Germany and Austria.

## Family typologies and parenting practices of polyamorous families

Every network that informed my research found its own way towards parenting. Nevertheless, the practices can be analytically divided into three forms of organized parenting: (a) poly-nuclear parenting (where only the biological and legal parents are involved in parenting practices, simulating a nuclear family), (b) hierarchical parenting (where at least three parents negotiate their positions as main or co-parents), and (c) egalitarian parenting (where at least three members of the polyamorous network claim to spend equal time with parenting).

Within the three parenting types, poly-nuclear parents are unique in that, at first glance, their parenting practices do not differ from normative family models. However, their relationships affect their family and parenting practices as well. Hierarchical family types consist of at least three parents that include one or two main parents and one or several co-parents. Egalitarian parenting networks include three or more main parents and sometimes also one or several co-parents. A detailed description of the following family practices will shed some light on how parenting is configured in these family types. From a new materialist perspective, parents and their environments are co-produced within practices that mark the boundaries between various types of parenting. In all of the following cases, we learn how the boundaries of non-normative family formations are established within a world that does not support their parenting practices.

### Poly-nuclear parents

This group of cases includes parents who live in a polyamorous relationship, but only two partners take part in parenting practices. The parents defined themselves as (cis) men and women with biological children. One or both parents have other relationships, but these partners are not involved in parenting practices. The characteristics of this family type are: (a) a determined separation of the private and the public realm, (b) the creation of separate spaces and times for additional relationships—I call it privacy from privacy—and (c) the privilege and burden of choosing whom to tell about the additional relationships.

The parents illustrate the relationship as an entity that belongs to the parents, which should not affect the children’s lives, even when they know about their parents’ additional relationships and even when they know the additional partners personally. A good example is the story of Jana, Jon, and James. Jana is married to Jon and they have two children. Jana also has a long-term intimate relationship with James. They meet once a month for a weekend. James also has a wife and children. Jana and James have never met their partner’s spouses or children. In this case, the polyamorous relationship is lived completely separately from the parenthood of all three members of the network.

“I usually try to explain that the one thing has nothing to do with the other thing … also emotionally it has nothing in common” (Jana, poly-nuclear parent)

The parents are very determined about keeping various spheres of their lives separate. From a new materialist perspective, however, this is a result of boundary making practices that are simultaneously building these spheres and their relationships (and their people). A public and two separate levels of private realms (a private family realm and a private relationship realm) are established. Parents are involved in practices that keep those two private realms physically, temporally, and emotionally apart.

These practices are also in place, when partners are known to the children. Leo’s partners attend dinners as friends of the parents, but the parents do not make something special out of visiting partners and do not describe them as partners to the children.

“There were visitors [for dinner], they were funny and the children were a part of it until they had to go to sleep.” (Leo, poly-nuclear parent)

While in the case of Jana, a distinction between private family life and private relationship time is established by traveling to another town (physically), on a specific weekend (temporally) that enables her to temporally separate the (emotional) connection to her family, in this case the temporal, physical, and emotional demarcation between private family life and private relationship time works within the same house, by sending the children to their room at a specific time to create spaces or times for relationships outside the nuclear family. Leo, who is married with children to Linda (both have additional partners), described a specific window of time in the evening that they called “parents’ free time.” Some days a week, the children are encouraged to retire to their rooms after dinner, which gives the parents time to either go out and meet a partner, have a partner over, or talk to partners on the phone. The parents made clear to their children that everybody wants and needs a space for privacy that should not concern the other members of the family. These requirements also needed physical space. Leo had the privilege to live in a house that provided the possibility for space and time apart.

“Then it was parents’ free time and the children went to their room. Whether they played or slept was their concern. And so they did their thing and we did our thing. The generous space [of the house] and the spatial layout made this easy.” (Leo, poly-nuclear parent)

“Parents’ free time” indicates that relationship time with someone, who is not a parent of the children, is a form of time off from family life. Therefore, the realm of private relationships works as another level of privacy. There is private family life and parents can have privacy from this non-public level too – privacy from privacy. While private family life is distinguished from public work life, it is never the less also the realm where care work (parenting and housekeeping) has to be taken up. The relationship realm is established as another level that separates an individual from its role as a public and family individual. The physical, temporal and subsequently emotional separation of the parenting and the relationship practices eventually produce humans that – most of the time – are either parents or partners. This also configures humans that are able to switch between these positions by moving through specific spaces (to a specific private room) at certain times (after the children are sent to bed).

The blurring of the established boundaries, for example, by switching to another family model, therefore also needs preparation and careful reflection. Bernhard, Berta, and Bianca also form the core of a polyamorous network. Every partner in this network has additional long- or short-term partnerships. Bernhard and Berta are married and they live together with their child. Bernhard meets Bianca once a week and Bianca attends Dinner at Bernhard and Berta’s “every other week.” Bernhard and Berta are unsure how to deal with Bianca’s role. Yet they also want to include her in their daughter’s life. Therefore, the future of Bianca’s role is unclear, as is this network’s family model. Bernhard and Berta can imagine Bianca as a co-parent:

“If Bianca becomes even closer to our family, she may be perceived as a kind of second mother by our child” (Bernhard, poly-nuclear parent)

This quote also indicates that if Bianca becomes a second mother, the demarcation between the realms of family and relationship blurs and the boundary that separates an individual from parenting and care work would collapse. Privacy from family life would not be possible anymore.

However, even when parenting and romantic relationships are treated as separate realms, most parents deem it necessary to tell their children at some point about their relationship model. Jana’s oldest daughter knows about her boyfriend. Jana informs her child in an age-appropriate way about this relationship and her daughter is rather unconcerned about her mother’s relationships outside the core family.

“I told my older daughter, who is rather mature for her age, that this is also about sex… and she said, yes, but it would be like saying you can only play chess with one person and with nobody else.” (Jana, poly-nuclear parent)

From her mother’s perspective, Jana’s daughter does not seem to have a problem with her mother’s weekends off. For Jana, being honest to her children in an age-appropriate way was very important. Her daughter is also old enough to decide whom to tell outside the family. Bernhard and Berta, who have a considerably younger daughter, are unsure if they should tell their daughter about Bianca’s role in their lives, because they are afraid of stigmatizing the child.

“How can I let such a small person live with this in this monogamous-oriented world, what if she tells? But should we keep it hidden?” (Bernhard, poly-nuclear parent)

Bernhard and Berta are in a dilemma. They would have liked to tell their daughter about Bianca from the very beginning because Bianca is a part of their lives, but they also feel threatened by the norms of monogamy.

Later in this article, I will show that not every network has the privilege of thinking about this decision. If necessary, poly-nuclear families have the advantage that they are passing as a nuclear family: they can act in a way that would fit the norm. Therefore, making the decision over and over again about who and when to tell is an important characteristic of this polyamorous family type, which is both a privilege and a burden. They can choose the picture others have of them, but at the same time they have to put work into maintaining that specific image. This can create considerable stress, but is also related to the privilege of private spaces configured by simulating norms and means to create physical spaces and times for additional partners. However, from a new materialist perspective, this is not the only reason for keeping this family model intact. In this family type, the realms of public, private family and private relationship life are carefully separated producing humans with specific responsibilities and agencies at a very specific time. People included in this family model have the possibility to establish a very specific form of privacy from family life on a regular basis. This level of privacy would collapse if another family model is taken up. Therefore, the boundaries of this family type do not allow the included people to transgress or demolish this boundary because it has produced people that also emotionally benefit from the specific private space and time.

### Hierarchical parenting

These parenting practices include more than two parents that put unequal time and effort into parenting. Parents differ between main and co-parents, which is either explicitly negotiated or established implicitly over time. The biological and/or legal parents are not necessarily the main parents who do the most care work and spend the most time with the child. Central characteristics of this family model are (a) a rather clear division of roles and tasks among main parents and co-parents and (b) the family is publicly visible as a multi-parent (queer) family.

Tori and Tina both have close ties to Thomas. All three wanted a child, but Thomas’ occupation includes a lot of traveling abroad. They then decided that Tori should have the child and Tina should be the legal parent. Tori and Tina got married. Thomas has no custody (and therefore also no financial obligation), but is involved as a co-parent. Tori and Tina make the main decisions and do the everyday care work. Thomas stays with Tori and Tina every few weeks and is then involved in the everyday tasks. When he is away, Thomas communicates with Tori, Tina, and their child by text and video chat. Their roles were negotiated at the beginning of parenthood and did not change much after that.

“It is not that complicated, it depends on the type. We are both types that are willing to compromise and also with the father, starting at the beginning, we talked about everything … I think it is no more complicated than most normal relationships, because we three are good at talking to each other, I think, after the span of three years where the project has been running.” (Tori, main parent).

Tori has been included in two phases, a phase of negotiation of a clear division of tasks (that is producing individuals who are able to communicate, reflect, and compromise) and a subsequent phase of routine, where these tasks are repeated without necessary re-negotiation (that is, producing individuals who follow up on their tasks without the need for reflection or communication about it).

In all cases, parents found themselves in routines that govern the time spent with children. For example, Onna is in a relationship with Olivia, who also has a relationship and a child with Olga. Olivia and Olga are the main parents, while Onna and the biological father, Otto, are co-parents. The child lives with Olivia or Olga most of the time, but stays with Olivia and Onna once or twice a week. The main parents are responsible for most of the caring tasks and for important decisions. Onna’s role in the child’s life involves playing with it and sometimes also doing some care work or picking the child up from school. However, such tasks are usually planned and orchestrated by the main parents. Onna compares her role to her perception of the role of a breadwinning father. She usually visits her girlfriend after a day of work and Olivia’s child is a major part of the beginning of this visit.

“He usually catches me in the hallway and then he wants me to play with him. I imagine that it’s like when a husband comes home from work at night and he is then allowed to bring his children to bed. This is what I imagine sometimes, but then it is also not like that, but we just play.” (Onna, co-parent)

For Onna, as well as for other co-parents, it is important that they have an independent relationship with the child. The main parents foster this independent relationship with the co-parents. Onna emphasizes that most of the time the life of everyday parenting seems to follow schedules and routines, such as school and work schedules. But there are days when she picks him up from school or when she visits, where she can live out her role as a partner playing with the child or reading to the child before bedtime. The practices of initial negotiation and subsequent routine carved out a parenting role for her that focuses on fun for her and the child. When decisions and care work are tasks of the main parents, playing games and entertaining the child are tasks left for co-parents. Onna perceives this as a transgression of her gender role, while from a new materialist perspective she is shaped as an individual, who is excluded from decisions and care work (and the specific relationship to the child these practices are producing) and who finds her relationship with the child in practices of entertainment for the child. These practices produce clear and separate realms of parenting tasks that are administered to specific individuals, which enable routinization, which in turn saves time for other things than negotiation, but also configures unequal parents.

Tori (see detailed description of her triad at the beginning of this chapter) also perceives her position as a transgression of norms, which is allowing her to break with demands that are placed upon them due to their social position or because of their gender. For Tori, who is parenting with main parent Tina and co-parent Thomas, the openness of their network was a relief.

“I especially like our situation, that we deviate from the norm, we have no pressure to fit any norm … I hear that from my siblings, okay, how do you have to be as a mother and father and what fits the norm and what doesn’t and I am so free, because I think okay, for example everybody knows I am untidy, but nobody cares, because whatever I do I am far from being the perfect normal woman and this is great, because I think we can do what we want and everybody thinks it is strange, and everybody says I wouldn’t want a third person involved and I like it, I have the freedom to do something else.” (Tori, main parent)

Even without Thomas in the picture, her relationship to Tina would not fit the norm (because she is married to a woman), so the decision about being open about their network was not that hard: She did not have the choice to comply with the norm (due to her sexual orientation). The family lives in an urban, liberal city, with a comparatively high level of redistribution of wealth. They work in liberal environments and lead an economically stable life. Experiences of discrimination have happened, but are not perceived as threatening. The unconventional relationship constellation gives Tori the freedom to also celebrate aspects of her personality that are deemed unwomanly, while she perceives her siblings, who live in heterosexual nuclear families, as restricted by norms. From a new materialist perspective, the practices of deviation seem to produce individuals that are less restricted by norms, given that an individual is embedded in a financially stable situation and liberal cultural environment. While poly-nuclear families talked about fearing stigma for their child, Tori and Tina are less concerned. They rather see an advantage for their child, who is also freed from some social norms.

“He doesn’t have to go to the perfect school and to the perfect kindergarten. That pressure, that the child has to fit, we don’t have that, because it is different.” (Tori, main parent)

Tori perceives a transmission of freedom of norms to her child, established by the special family situation she, Tina, and Thomas created together. Her child does not need to fit in kindergarten or school (hence fit gender or behavioral norms), nor are Tori and Tina in need to find the perfect place for her. Central European public daycare and schools are usually reasonably priced or free and of high quality. Highly educated parents nevertheless feel the pressure to choose the optimal education for their children. Tori emphasizes, she does not feel this pressure. She is rather confident that her child will turn out very well without the need to optimize every environment she will be in. It is important to Tori that she and her co-parents create a supporting environment at home and also that the child has the freedom to be as she wants to be. The parents’ non-normative relationship helps support this. From a new materialist perspective, these practices could configurate a space and time that allows the production of new norm-transgressing individuals.

This section illustrates, how in specific environments hierarchical parenting can be related to a transgression of norms way beyond norms that regulate gendered practices of parenting. Multi-parent practices that are visibly queer may configurate spaces and times that include humans who are not connected to predetermined tasks, roles or behaviors. A clear division of tasks and responsibilities among main parents and co-parents produces inequalities between the parents, but also routinization, which shifts time from negotiation to other tasks or free time for each family member.

### Egalitarian parenting

In egalitarian parenting families at least three parents share an equal amount of care work and responsibility for the child. These networks can also include less involved co-parents and partners who do not parent. The central characteristics of this parenting type are (a) a thorough and repeated negotiation of the division of tasks, (b) a related transgression of gender roles and norms, and (c) less stress and pressure for individual parents because parenting is shared.

While in hierarchical parenting networks tasks are initially negotiated and then routinized, which is producing rather stable individual roles with fixed tasks, parents in egalitarian networks perceive themselves as constantly negotiating everything: their roles as parents as well as their own identities. Roger, who is parenting equally with Rita and Robert, imagines some kind of package deal (a house and a heterosexual nuclear family) that two adult-parents want or follow. He emphasizes that in his form of living, every relationship must be negotiated.

“There is no complete package anymore. There is, if you kiss somebody, you have to marry and have children and buy a house and you spend all your free time with each other, this is a polemic exaggeration, but there everything belongs to a package and it is known what belongs to it. We do not live that simply.” (Roger, egalitarian parent)

Not living that “simply” means that considerably more work has to be put into negotiating even such fundamental issues as who is a parent and how large the parental involvement of every partner will be. Roger is married to Rita and Rita also has another partner, Robert. Roger also has another partner, who is not involved in parenting. Robert lives in a flat above Roger and Rita. The spatial closeness of his apartment allows him to join Roger and Rita in the mornings in order to be involved in everyday tasks and to be near to his child. It is important to Roger that Robert is an equal parent to his child and that all tasks and responsibilities are shared with him.

All parents, who identify as egalitarian parents emphasize that their family members require good negotiation skills and the ability to talk things through and to compromise. Every family has a different approach to negotiating time and tasks. Some networks sit down every week and plan the tasks for the upcoming week, while others manage their time in an ongoing daily routine of talking in person, completing joint online calendars and negotiating through instant messengers. Fiona is in a relationship with Flora and Fabian. All three are main parents in addition to Felix, a former partner and the child’s biological and legal father. Each partner and main parent also has other long- or short-term partnerships. To negotiate their time, they rely heavily on digital means of communication:

“This, for example, is our joint calendar [shows something on the display of her smartphone]. This is my calendar, this is Flora’s calendar, this is our joint calendar, this is Fabian’s calendar, and this is when the child is with us.” (Fiona, egalitarian parent)

For the non-parenting partners of Fiona, Flora, Fabian, and Felix, it seems implicitly evident that they have no influence on parenting questions. They are involved solely in scheduling negotiations. The caring tasks for their child are negotiated electronically and on an everyday basis when partners meet face to face. In contrast, Iwan, who shares parenting with Irene and Ida, is used to fixing schedules and tasks once a week. In addition to these weekly sit-downs, additional meetings can be arranged if something unexpected comes up.

“We sit down again and again, with the weeks ahead, dealing with what’s to come next… we want [our child] to have regularities in his life, so he knows what he can expect, when we pick him up, what his tasks are, our rules.” (Iwan, egalitarian parent)

The aim, for this triad, is to provide a relatively fixed setting for their child. Iwan, Irene, and Ida prefer to agree on a long-term fixed schedule, detailing when and by whom their son is picked up from school, how they deal with decisions and what the important rules are, which everybody has to stick to. All three parents work part-time (also with fixed schedules) and they try to divide their time off work equally between caring tasks and free time. Rather than a strict and predefined division of tasks, the families established an individual plan for repeated thorough and quick negotiations. This process uses up a lot of time and means a lot of work, but also creates the freedom to deviate from traditional parental roles.

In addition, all parents point to the increased time and attention that their child receives. Children can be demanding and stressful. Parents are relieved to share the stressful tasks, while the child has more attachment figures and quality time with parents. Flora emphasized that the first months with a newborn child can be especially overwhelming. The network of parents reduced stress and allowed overwhelmed parents to have some time off. For Fiona, the biological mother, this also meant time to return to work early and not having to rely on traditional divisions of work. This is perceived as an advantage for their chlid, because one parent is almost always available.

“When one of us was overwhelmed with the child, they just gave her to the next person.” (Flora, egalitarian parent)

“She has so many attachment figures, she has no fear of separation… and for me, this mother thing, not going out of the house for the first three years [after the birth of the child], this didn’t happen.” (Fiona, egalitarian parent)

Although all parents have to deal with the legal regulations regarding custody, which only applies to two parents, the parents stress that their children do not belong to the legal parents alone. The tenor in all interviews is that the polyamorous parents cannot imagine how “only two” parents are able to master the challenges of parenthood.

“the child demands a lot of attention, the existence of this poly-network is an unbelievable relief.” (Roger, equal parent)

Although negotiations of the division of work takes up a lot of time, the sharing of parenthood is perceived a major relief of responsibility and stress that results in free time for each individual.

From a new materialist perspective, the practices of parenthood produce individuals that are caring and have responsibilities in multiple ways. A group of people is shaped that carries the tasks of parenthood together. Individual preferences for specific care work are less recognized (because everybody should know and do every task on a regular basis). Further, people have more time for themselves or individual tasks because the parenting and housekeeping tasks are shared among more people. Because everybody is asked to know and do every task, there are no roles attached do a bundle of specific tasks (such as in traditional family models, or in hierarchical and poly-nuclear settings), therefore also gender, gender-conformity, and gender norms are less discussed, providing space for individuals that are not mothers, nor fathers (but just individuals or explicitly non-binary parents).

This section discussed how egalitarian networks share and negotiate work constantly, which also results in decreased perception of stress and increased freedom for each involved parent. However, are there situations where hierarchical and egalitarian parents also emphasize traditional structures? The next section will shed a light on this question.

### Parents pushed and pulled through the various parenting practices

A deeper (new materialist) analysis of the same data shows a fluidity of these parenting models. Depending on context and situation parents are pushed and pulled into different models of parenting and specific models of dividing care. In this part of the article, I explore the contexts and situations that push and pull non-monogamous parents into specific unintended models of parenting. Further, these processes have implications for the question if practices of collective parenting transgress and subvert established social structures (e.g., heteronormativity or economic inequality). This second part of analysis shows that collective parenting can be subversive and affirmative at once, depending on the complex entanglement with situational contexts.

While the above described parenting types are stable for a longer time, a closer analysis of the interviews show that, in specific situations, all parents deviate from the style of parenting they identify with. For example, Roger describes a family gathering at Rita’s parents. Relatives do not understand their concept of family and parenting and seem to believe that Roger is the ex-husband and Robert is the new partner. Even Rogers’ partner, who is not involved in parenting practices, fits in this picture. The relatives perceive the polyamorous family in the light of serial monogamy and heterosexual relationships and cannot perceive anything outside of these concepts. Rita became tired of explaining; therefore, they just let their relatives believe what they want. Consequently, they become a (poly-nuclear) family that is simulating a heterosexual partnership. It is important that from a new materialist perspective within these hours they *are* a serially monogamous couple sharing parenthood. They enact this family model and their (secret) egalitarian polyamorous relationship does not come to matter in this moment. It is non-existent in this moment and is re-activated on the way home by changing (material and discursive) contexts. They become a part of practices that rebuild the egalitarian parenting model on their way home from this family gathering: for example, in the car, when they discuss and reflect the family event and then the upcoming week and family matters and when they come home to their specific living situation (all living in the same house across two flats sharing all the work) where they enact their usual family routine again. Therefore, when they go to a family gathering they travel between two worlds in which they *are* two different forms of family.

In a similar way, Daniela explains how a transition from the primary to the secondary school system changed the networks’ presentation toward the school. For the new school, Daniel and his wife appear as a heterosexual couple. The teachers are not that interested in contact to parents anymore and an occasion to explain never rose.

“In the old school, they knew, we are some kind of new patchwork family. That thing with the school, it was clear to me that they [the biological parents] do that.” (Daniela, co-parent)

Again the network becomes invisible and resembles a poly-nuclear parenting style, in this situation. The school, not interested in getting to know the living situation more closely, resembles the experiences of parents with schools Pallotta-Chiarolli (2010) describes in her work with the concept of border families. Parents are categorized along well-known family models (heterosexual couple, step-families, or gay couples) and other family models have a hard time becoming part of the picture (Pallotta-Chiarolli, 2010). From a new materialist perspective, when Daniel and his wife deal with school matters, they *are* a married heterosexual couple solely responsible for their child. Daniela’s co-parenthood does not appear in this picture, while she is included in the weekly (non-school) routines. The transfer from one school to another drew a new and clear boundary within the family and Daniela is excluded from some parenting responsibilities (while she was included in the previous school). Other weekly routines, where school issues do not matter, for example, Daniela routinely spending time with him and executing tasks, such as taking him to extracurricular activities, rebuild the co-parental family model and its boundaries.

There are also situations where more open family models are built: Leo, a poly-nuclear parent, is rather open to his environment about his relationships. His kids are now grown and know about their parents’ additional relationships. Family gatherings, a family wedding, or a birthday party can include the non-parent partners and old and new primary partners. In this situation, the queerness of the network becomes publicly visible being patchwork and polyamorous at the same time. The boundaries of the family are not set as clear as intended by the parents and the division between poly-nuclear family, additional partners, and former partners diminishes. All involved people *are* a family in this situation with a specific relationship to the children. The poly-nuclear parenting model is reestablished through the continuous contrasting routines described in the section above, when time with children is again clearly separated from time with non-parental partners, when, for example, the parents invite their now grown children on separate nights to dinner.

Especially when (cis) men and women are involved, parents experience situations beyond their agency, when they are pulled into a heterosexual world with specific gender roles, which creates invisibility for polyfamilies and maintains traditional structures. But also those, who set strict boundaries between parenthood and polyamorous relationships enter spaces where these demarcations become less clear. I derive the following findings from the everyday processes described in this chapter: Even the economically stable and highly educated parents in this sample cannot control that they always act according to their identity. There are spaces, where intention and identity are not operable and there are times and spaces where co-parenting and egalitarian parenting family models can become visible as the family model they intend to be. Further investigations of the material discursive conditions that facilitate the latter are necessary.

## Conclusion

Polyamorous networks with children differ in their approach toward parenting. I found three major sets of practices that form three family types: poly-nuclear families, hierarchically parented families, and equally parented families. An important characteristic of poly-nuclear families is a clear boundary between family time and private time for each partner. While family is often defined as belonging to the realm of privacy, the family members enact another level of privacy by arranging times to retreat from the family. A characteristic of this family model was also the privilege to choose the visibility of the polyamorous network carefully and to pass as a heterosexual nuclear family.

For families that divide parenting hierarchically between main and co-parents, this question was less prevalent because the parents’ relationships were either always visible as queer (e.g., two legal mothers) or they made efforts to increase their visibility. Within the comparatively liberal social market economy in parts of Germany and Austria (universal healthcare, free public schools, and liberal communities), the non-normative relationship is further perceived as a relief and freedom from gender norms and values. In general, parents are often under pressure to provide optimal education for their children, also by employing strategies to optimize parenting practices (Christopher, 2012), which can decrease parents’ wellbeing (Rizzo et al., 2012). In my findings, being visible as queer resulted in letting go other norms of optimization as well, which decreased pressure on parents and in turn increased their wellbeing. This privileged position may not be intelligible in more conservative or economically more liberal contexts.

Equally parented families put a lot of work into their negotiation of their divisions of tasks. There are no predefined roles or tasks for each parenting partner. Therefore, individuals explicitly negotiate their tasks and time with the child and pick up certain tasks that specific arrangements (work schedules and visiting times) allow for. The data hints at increased time for and attention to children and reduced levels of stress for the parents, as well as increased gender equality due to the fact that there are no predefined female or male roles. The family members in this study present themselves as highly skilled in negotiating roles and tasks, when no predefined norms and examples are available. However, it has to be considered that the sample for this project consisted of individuals with rather high social and cultural capital.

Finally, a second glance indicates that also hierarchical and equal parenting networks are situationally pushed into different roles and position, resembling traditional nuclear families and thus unintentionally maintaining traditional structures. From a new materialist perspective, these situations are an entanglement of situational structures, norms, discourses, bodies, processes, and so on that cannot be controlled by the individual. The parent’s self-identifications are rendered negligible in these situations, forcing them into practices that are re-defining the boundaries of normative family standards they usually want to deviate from. I conclude from these processes that self-identifications need specific contexts that provide the agency to the parents to act accordingly. Otherwise, everyday public practices produce parents that maintain traditional structures regardless of their intentions and identities.
